# Analysis of the Molecular Networks in Androgen Dependent and Independent Prostate Cancer Revealed Fragile and Robust Subsystems

**DOI:** 10.1371/journal.pone.0008864

**Published:** 2010-01-28

**Authors:** Ryan Tasseff, Satyaprakash Nayak, Saniya Salim, Poorvi Kaushik, Noreen Rizvi, Jeffrey D. Varner

**Affiliations:** 1 School of Chemical and Biomolecular Engineering, Cornell University, Ithaca, New York, United States of America; 2 School of Biomedical Engineering, Cornell University, Ithaca, New York, United States of America; Keio University, Japan

## Abstract

Androgen ablation therapy is currently the primary treatment for metastatic prostate cancer. Unfortunately, in nearly all cases, androgen ablation fails to permanently arrest cancer progression. As androgens like testosterone are withdrawn, prostate cancer cells lose their androgen sensitivity and begin to proliferate without hormone growth factors. In this study, we constructed and analyzed a mathematical model of the integration between hormone growth factor signaling, androgen receptor activation, and the expression of cyclin D and Prostate-Specific Antigen in human LNCaP prostate adenocarcinoma cells. The objective of the study was to investigate which signaling systems were important in the loss of androgen dependence. The model was formulated as a set of ordinary differential equations which described 212 species and 384 interactions, including both the mRNA and protein levels for key species. An ensemble approach was chosen to constrain model parameters and to estimate the impact of parametric uncertainty on model predictions. Model parameters were identified using 14 steady-state and dynamic LNCaP data sets taken from literature sources. Alterations in the rate of Prostatic Acid Phosphatase expression was sufficient to capture varying levels of androgen dependence. Analysis of the model provided insight into the importance of network components as a function of androgen dependence. The importance of androgen receptor availability and the MAPK/Akt signaling axes was independent of androgen status. Interestingly, androgen receptor availability was important even in androgen-independent LNCaP cells. Translation became progressively more important in androgen-independent LNCaP cells. Further analysis suggested a positive synergy between the MAPK and Akt signaling axes and the translation of key proliferative markers like cyclin D in androgen-independent cells. Taken together, the results support the targeting of both the Akt and MAPK pathways. Moreover, the analysis suggested that direct targeting of the translational machinery, specifically eIF4E, could be efficacious in androgen-independent prostate cancers.

## Introduction

Prostate cancer is the most common cancer in men and the second leading cause of cancer-related death in the United States [1]. It has been known since the 1940s that androgens, such as testosterone, are required for prostate cancer growth [2]. Accordingly, androgen ablation in combination with radiation or traditional chemotherapy remains the primary non-surgical treatment for androgen-dependent prostate cancer. Androgen ablation initially leads to decreased tumor growth and reduced secretion of biomarkers such as Prostate Specific Antigen (PSA) [3]–[5]. However, in nearly all cases androgen ablation fails to permanently arrest cancer progression. As testosterone is withdrawn, malfunctioning prostate cells lose their sensitivity to androgen and begin to proliferate without hormone growth factor signals. These testosterone insensitive cells can then lead to Androgen-Independent Prostate Cancer (AIPC) [6]. The AIPC phenotype is closely related to metastasis and decreased survival. Unfortunately, current treatments for metastatic AIPC have demonstrated only modest survival advantages [7]. Thus, an effective therepy for metastatic AIPC represents an unmet medical need and an ideal target for systems biology.

AIPC is characterized by androgen action in the absence of androgen stimulation. At the core of androgen action is the regulation of Androgen Receptor (AR) by hormones such as testosterone. AR is a cytosolic steroid hormone receptor belonging to the superfamily of ligand activated transcription factors. Other members of this family include Vitamin A/D, estrogen, progesterone and thyroid hormone receptors [8], [9]. In healthy prostate epithelial cells, androgens activate AR and drive an AR-dependent gene expression program. Sexual androgens such as testosterone typically circulate in the blood, bound to proteins such as the Sex Hormone Binding Globulin (SHBG) protein. Free testosterone enters prostate cells where the 5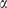-reductase enzyme converts it to activated dihydrotestosterone (DHT) [10]. Both cytosolic testosterone and DHT can bind AR, however DHT has a higher affinity for AR. Binding of DHT to AR promotes cytosolic AR activation and the translocation of activated AR to the nucleus. Nuclear AR drives the expression of target genes including PSA by binding to AR-responsive promoter elements [11], [12]. Because of its ligand dependence, one would expect AR activation and AR-driven gene expression to be absent without hormone stimulation. However, AIPC often has higher PSA expression and increased cell-proliferation compared to its androgen-dependent counterpart even without stimulation [13], [14].

AIPC's increased proliferation and PSA secretion in the absence of androgen suggests a failure in the regulation of androgen receptor activation. Feldman and Feldman reviewed several possible AR regulatory pathways perhaps responsible for androgen action in the absence of hormone stimulation [15]. One hypothesis, referred to as the hypersensitivity pathway, suggests that AR may be more sensitive to androgen in AIPC. This would allow AR activation and AR-driven gene expression at much lower levels of extracellular testosterone signals. Another hypothesis, referred to as the promiscuous pathway, suggests that AR can be activated by non-androgen antagonists. A third hypothesis, explored here, suggests that AR can be activated by other pathways, for example, the Mitogen Activated Protein Kinase (MAPK) cascade. Several studies support this cross-talk hypothesis, sometimes referred to as the outlaw pathway. Culig *et al.* showed in DU-145 human prostatic tumor cells that growth factors e.g., IGF-I, KGF, and EGF could drive AR activation without androgen [16]. Nazareth and Weigel showed in human prostate PC-3 cells that AR could also be activated by the protein kinase A activator, forskolin in the absence of androgen [17]. Other studies have suggested a connection between Her2 induced activation of the primary MAPK cascade and AR activation [18]. For example, Her2 overexpression was positively correlated with diminished sensitivity to androgen ablation, increased AR dependent PSA expression, increased AR activation, increased tumor mass and shortened tumor latency [14], [18]–[20]. Thus, one would expect regulators of Her2 activation, for example the different forms of the 100 kDa glycoprotein Prostatic Acid Phosphatase (PAcP), could be important factors in androgen dependence and tumor grade [21]–[26]. Intracellular PAcP (cPAcP) whose expression is AR responsive, downregulates Her2 by dephosphorylation. On the other had, secreted PAcP (sPAcP) promotes modest Her2 activation by an unknown mechanism [26].

## Results

**Figure 1:**
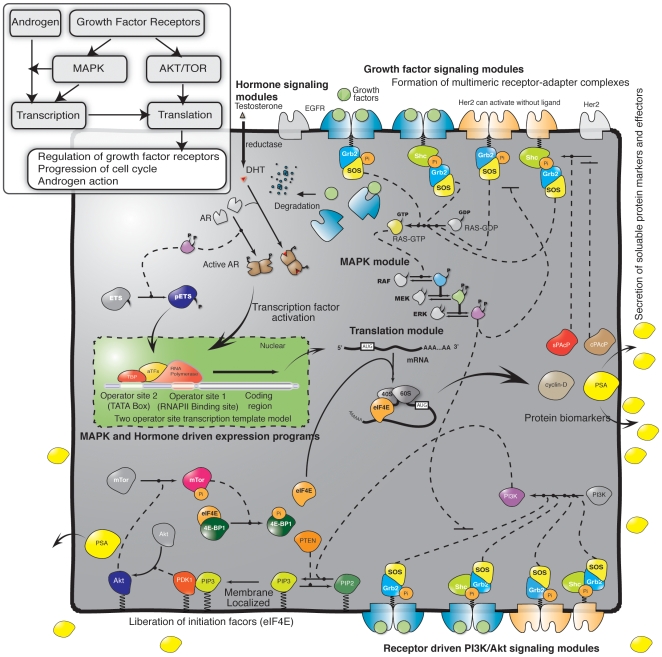
Schematic overview of the interaction network used in modeling the androgen response in prostate epithelial cells. The model architecture was formulated by aggregating molecular modules into a single network (see insert for high level details). The model describes growth factor and hormone induced expression of cyclin D, PSA and the two forms of PAcP. The complete list of molecular interactions that comprise the model (along with kinetic parameter values) are given in Table S1.

The objective of this study was to determine which signaling components were important in AI versus AD LNCaP cells. Toward this objective, we constructed and analyzed a mechanistic mathematical model of the androgen response of three different LNCaP prostate adenocarcinoma sub-lines. We investigated MAPK-dependent outlaw activation of AR in AD (C-33), mid-range (C-51) and AI (C-81) LNCaP cells [13], [27]. Our network model included: nuclear hormone and transmembrane growth factor receptor activation; transcriptional activity via the MAPK subsystem [28]–[30] together with outlaw activation of AR via MAPK [15], [18]; PI3K/AKT/TOR mediated translation initiation [31], [32]; the transcriptional and translational regulation of PSA, cyclin D and PAcP expression [14], [20]; and the regulation of Her2 activity by PAcP [26] (Fig. 1). The network described 212 species and 384 interactions (Table S1). Transcription and translation were modeled using elementary reactions based on literature (supplemental materials). Constitutive and regulated expression of PSA, cyclin D and the two forms of PAcP were considered in the model. The *total* level of all other model proteins was constant. We modeled the molecular interactions using mass action kinetic processes within an ordinary differential equation (ODE) framework. ODEs are a common method of modeling biological pathways and have been used to model a range of signal transduction processes [29], [33]–[41]. Mass action kinetics have also been used extensively, for example, to model receptor tyrosine kinase signaling [41], blood coagulation [39], pain networks [40] or Toll like receptor signaling [42], [43]. They have also been a key component in the success of perturbation-response approaches which have shown that simple linear rules often govern the response behavior of biological networks [44]. The ODE model was deterministic and captured only population averaged behavior. While we assumed spatial homogeneity, we differentiated between cytosolic and membrane localized processes. We used mass-action kinetics to describe the rate of each molecular interaction. Thus, the 384 kinetic model parameters were mainly association, dissociation or catalytic rate constants. With one exception, model parameters were estimated and validated using LNCaP training data taken from literature sources (Table S2). However, we were unable to estimate unique model parameters. Instead, we estimated a family or *ensemble* of parameters that was consistent with the training data. The ensemble allowed us to estimate the model uncertainty associated with the many poorly characterized parameters. We analyzed the model ensemble to better understand which architectural features were important in androgen dependent versus independence cells.

### Estimating the Ensemble of Prostate Model Parameters

Signal transduction models often exhibit complex behavior [45]–[48]. It is often not possible to identify model parameters, even with extensive training data [49]. Thus, despite identification standards [50] and the integration of model identification with experimental design [51], parameter estimation remains challenging. In this study, an *ensemble* of plausible model parameters was estimated from AI and AD LNCaP sub-clones. Ensemble approaches have successfully addressed uncertainty in systems biology and other fields like weather prediction [40], [52]–[55]. Their central value is the ability to constrain model predictions despite uncertainty. For example, Sethna and coworkers showed in a model of growth factor signaling that predictions were possible using ensembles despite incomplete parameter information (sometimes only order of magnitude estimates) [46]. They further showed that model ensembles were predictive using many different mathematical models [56].

**Figure 2:**
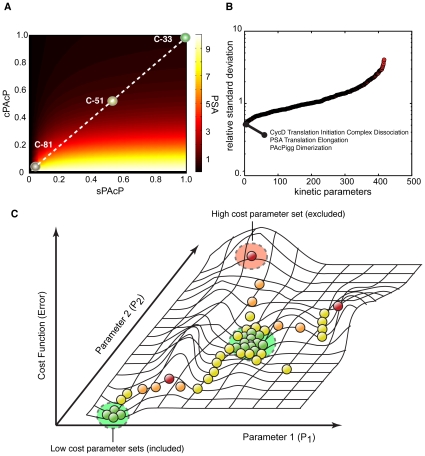
Identification and properties of the prostate model ensemble. **A**: Steady state PSA level as a function of cPAcP and sPAcP expression. The circles represent the values used to model the C-51 and C-81 LNCaP clones. All values are relative to C-33. **B**: Coefficient of Variation (CV; standard deviation of a parameter relative to its mean value) for the parameter ensemble used in this study. A small CV suggested a parameter was tightly constrained by the training data used for model identification. The parameters with the three smallest CVs are listed. **C**: Parameter identification strategy. Multiple monte-carlo trajectories were used to randomly explore parameter space. The simulation error and the correlation between parameter sets was used to generate the family of parameter sets used in the simulation study.

The 420 unknown model parameters (384 kinetic constants and 36 non-zero initial conditions) were estimated using 14 time-series and steady-state training sets taken from literature sources (Table S2). The parameter identification procedure used a maximum likelihood random-walk strategy with a correlation constraint to identify a diverse family of likely parameter sets (Fig. 2C). We generated 3210 possible parameter sets and selected 107 of these for inclusion in the final ensemble. The selection was made to minimize the correlation between possible sets (materials and methods). The majority of parameters had a Coefficient of Variation (CV) of greater than 100%. Thus, although the model qualitatively recapitulated the training data, many of the parameters were poorly constrained (Fig. 2B). However, parameters involved with key features such as cyclin-D and PSA expression were relatively well constrained (CV
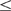
50%). The low deviation of these parameters could be attributed to the abundance of PSA/cyclin D training data. Alternatively, it may suggest that these mechanisms had a large impact on model behavior. A single network structure described both Androgen Dependent (AD) and Androgen Independent (AI) training data with only two experimentally justified parameter changes. The parameters controlling the expression rate of cellular PAcP (cPAcP) and secreted PAcP (sPAcP) were reduced by a factor of 0.01 and 0.5, respectively, for the C-81 and C-51 cell-lines compared to C-33 (Fig. 2A). The PAcP expression scaling factors were chosen to correspond with measured steady-state PAcP expression ratios for the different cell-lines [57]. The kinetic parameters and non-zero initial conditions for C-33 are given in Table S1 and Table S3, respectively.

### The Ensemble of AI/AD Lncap Models Recapitulated Androgen Action and the Activity of the Outlaw Pathway

**Figure 3:**
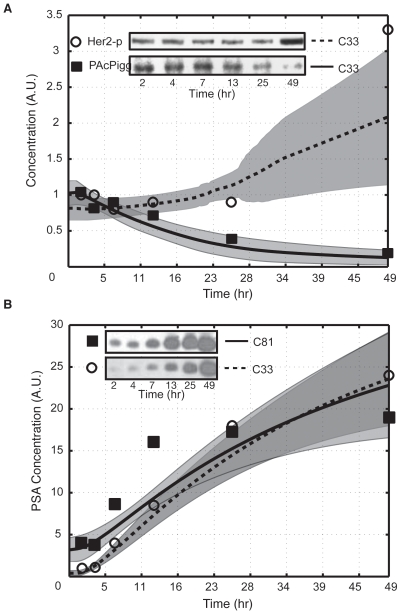
Simulation results for the addition of 10nm DHT at 1 hour to C-33 and C-81 LNCaP clones. **A**: Her2 phosphoralation (circles) and cPAcP expression (squares) for C-33 cells following the addition of DHT. Experimental data reproduced from Meng *et al.* [20]. **B**: PSA expression following the addition of DHT to C-81 (squares) and C-33 (circles) LNCaP clones. Experimental data reproduced from Lee *et al.* [14]. The shaded region in each plot denotes one standard deviation centered about the ensemble mean (line).

**Figure 4:**
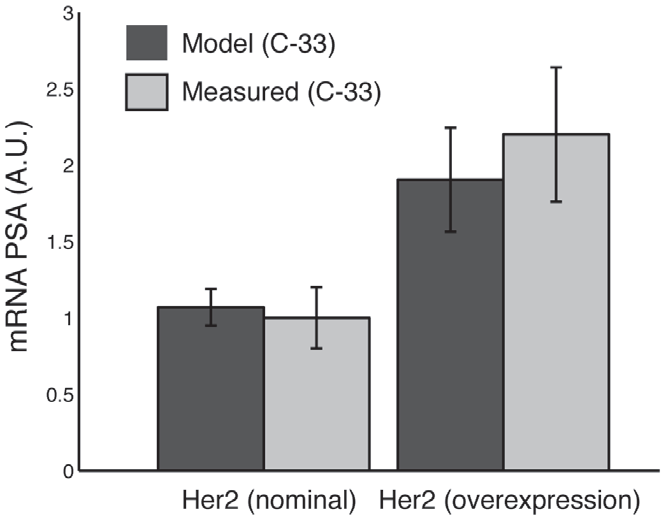
Simulated PSA mRNA levels in C-33 cells with and without Her2 overexpression. Her2 overexpression was modeled as a 50% increase in the expression rate of Her2. Bars denote the mean PSA mRNA level over the parameter ensemble while error bars denote one ensemble standard deviation. The experimental PSA mRNA data was adapted (replotted) from [18].

AR can be activated by both hormone dependent and independent pathways. In this study, we considered both the traditional hormone dependent and MAPK mediated AR activation. We selected training data sets to constrain each mode of AR activation and the subsequent AR-driven gene expression program. The data of Lee *et al.*, was used to constrain the relationship between PSA expression and AR activation in AI and AD cells [14]. Activated AR was modeled as both a transcriptional activator of PSA expression [58] and a transcriptional represser of PAcP expression [20]. The model recapitulated the qualitative features of PSA expression at the protein level for C-81 and C-33 (Fig. 3B). Additionally, the basal and increased level of PSA mRNA following Her2 overexpression in C-33 was also well described (Fig. 4). The PSA mRNA data was taken from a separate LNCaP study [18]. The C-33 simulations recapitulated the observed lower PSA expression (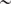
4 fold) compared to C-81 in the absence of androgen (Fig. 3B, initial point). Following DHT stimulation (10nM at t = 1 hr) PSA expression increased for both clones. However, the increase was more significant for C-33 (Fig. 3B). The study of Meng *et al.* was used to constrain the relationship between AR activation and PAcP expression [20]. The addition of DHT to C-33 cells decreased PAcP expression and increased Her2 phosphorylation (Fig. 3A).

**Figure 5:**
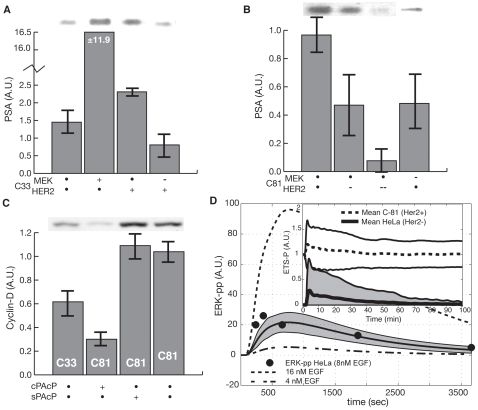
Simulation results for key species under androgen free conditions. **A**: Effect of HER2 and MEK overexpression on LNCaP C-33 steady state PSA levels. The inhibition of MEK blocks the effect HER2 overexpression. Experimental data adapted from Lee *et al.* [14]. **B**: Effect of HER2 and MEK inhibition on LNCaP C-33 steady state PSA levels. The inhibition of either HER2 or MEK blocks high AIPC PSA levels. Experimental data adapted from Lee *et al.* [14]. **C**: Effect of PAcP isoforms on LNCaP steady state cyclin D levels. Experimental data adapted from Lingappa and coworkers (Prosetta Corporation, unpublished data). **D**: Transient activation of ERK via ligand dependent EGF signaling (8nM EGF at t = 60s) in HeLa cells. The HeLa data was reproduced from [30]. Inset: Simulated phosphorylated ETS (ETSp) levels following the addition of 8nM EGF in the presence and absence of Her2. Her2 activation drives a sustained MAPK signal which in turns sustained ETS activation. The shaded region denotes one standard deviation centered about the ensemble mean (line).

**Figure 6:**
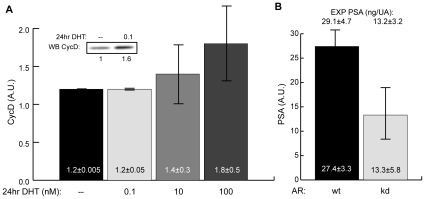
Independent model predictions versus experimental observations. **A** Ensemble prediction of cyclin D expression following the addition of DHT at 1 hour to C-33 clones. The ensemble predicted a dose dependent increase of cyclin D at 24 hours after DHT addition. Experimental data was adapted from Barnes-Ellerbe *et al.* [60]. **B** Predicted effect of an AR knockdown on PSA expression following the addition of androgen at 1 hour to C-33 wild-type and C-33 AR knock-down clones. The ensemble predicted an approximate 50% decrease in androgen stimulated PSA expression due to AR knock-down 72 hours after treatment. Experimental data was reported by Eder *et al.* [61]. The error bar denotes one standard deviation centered about the ensemble mean.

The model recapitulated the positive feedback between Her2 induced MAPK activation and androgen action. Several studies have demonstrated that MAPK can activate AR in the absence of hormone stimulation. Activated AR transcriptionally down-regulates cPAcP expression which in turn increases Her2 activation. Both Her2 dimerization along with the traditional EGFR-growth factor pathway can activate MAPK, leading to a positive feedback loop. However, typical growth factor induced MAPK activation is transient whereas de-regulated Her2 induced MAPK activation is persistent. The MAPK module in the model described both activation pathways. Growth factor dependent MAPK activation was constrained by dynamic measurements of phosphorylated ERK (ERKpp) levels following stimulation of EGFR with 8nM EGF (Fig. 5D). The EGF induced ERKpp data was taken from HeLa cells [30]. However, we expect transient EGF-induced MAPK activation in LNCaP cells will be qualitatively similar to HeLa given the conserved nature of mitogenic signaling. We constrained Her2 induced MAPK activation using cyclin D protein expression data in C-33 and C-81 cells without androgen following PAcP expression (Fig. 5C). Cyclin D expression was coupled to ERK through the ETS and AP1 transcription factors, both of which activate cyclin D expression [59]. Her2 induced MAPK activation led to a persistent ETSp signal compared to ETS activation following EGFR-induced MAPK activation (Fig. 5D, inset). Nominally, C-33 cells have lower cyclin D expression compared to C-81 (Fig. 5C, lane 1 and 4). The difference in cyclin D expression between C-33 and C-81 cells was qualitatively consistent with increased C-81 proliferation [13]. While the expression of cPAcP in C-81 reduced cyclin D levels (Fig. 5C, lane 2), sPAcP expression resulted in no change (Fig. 5C, lane 3). Furthermore, the model *predicted* a dose dependent increase in C-33 cyclin D levels 24 hours after addition of DHT (Fig. 6A). Although the cyclin D increase is only notable in response to high levels of DHT (10 or 100nM) the prediction is qualitatively consistent with experimental data *not* included in the ensemble calculations [60].

To further constrain the relationship between MAPK, Her2 and AR activation, we used the Her2 perturbation study of Lee *et al.* [14] in the ensemble calculations. Because the perturbation magnitudes were not reported, we assumed 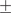
50% for all changes. Where possible, this assumption was validated by analyzing the corresponding Western blots using the GelEval software package (v1.22, Frog Dance Software). The 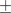
50% perturbation magnitude was approximately consistent with the published blots. A 50% increase in Her2 led to an approximately 50% increase in PSA expression in C-33 without androgen (Fig. 5A, lanes 1 and 3). While a 50% decrease in Her2 in C-81 led to a similar decrease in PSA secretion (Fig. 5B, lanes 1 and 2). Further disruption of Her2 effectively blocked PSA expression in C-81 without androgen (Fig. 5B, lane 3). A 50% reduction of MEK, one of the three primary protein kinases in MAPK, resulted in reduced PSA expression in C-81 (Fig. 5B, lane 4). While a 50% increase of MEK in C-33 increased PSA expression by 5-fold (Fig. 5A, lane 2). The combination of MEK inhibition and Her2 activation (50% increase in Her2 and a 50% decrease in MEK) decreased PSA expression in C-33 (Fig. 5A, lane 4). Furthermore, the model *predicted* an increase in C-33 PSA levels 72 hours after a 2nM addition of the androgen testosterone. Simulations performed with 10% of the AR initial condition predicted an approximate 50% decrease in testosterone stimulated PSA (Fig. 6B). The reduced PSA levels are consistent with reported experimental data on AR antisense knock-downs in androgen dependent LNCaP cells [61]. This data was *not* included in the ensemble calculations. Taken together, the model replicated qualitative features of the relationship between MAPK, AR activation and androgen action. In addition, the qualitative agreement between model and experiments for PSA and cyclin D expression suggested that the transcription and translation subsystem models were operating correctly.

### Sensitivity and Robustness Analysis Revealed Key Subsystems in AI and AD Cells

**Figure 7:**
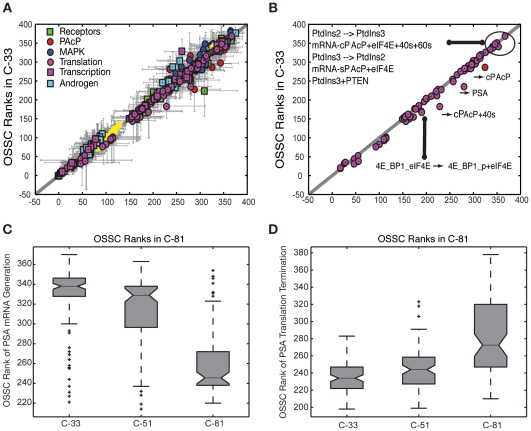
Sensitivity analysis of the model parameters. **A**: Comparison of the mean OSSC parameter ranks for the C-33 and C-81 LNCaP models. Large ranks indicate fragility. Points left of the 45 line are more important in C-33, while shifts to the right show increased importance in C-81. Points are organized by biological function. **B**: Comparison of the mean OSSC parameter ranks for translation mechanisms (including the role of Akt signaling in translation initiation) in C-33 versus C-81 LNCaP clones. The error bars indicate one standard deviation centered about the mean ensemble value. **C**: The final mechanism in PSA transcription becomes increasingly more robust w.r.t cancer aggressiveness, as indicated by a significant reduction in mean OSSC Rank. **D**: The final mechanism in PSA translation (translation termination) was increasingly fragile w.r.t cancer aggressiveness, as indicated by a significant increase in mean OSSC rank. The results indicate a shift in the bottle neck for generation of PSA from transcription to translation as prostate cancer cells lose their androgen dependence. The top and bottom of each box denote the 25th and 75th percentile of the OSSC rank over the parameter ensemble. The center line denotes the median value. Whiskers show the furthest observations and black crosses indicate outliers.

Sensitivity analysis identified interactions important in C-33, C-51 and C-81 cells (Fig. 7 and Table S4). We calculated overall State Sensitivity Coefficients (OSSCs) for the three LNCaP clones over the parameter ensemble (materials and methods). The OSSC values were ranked-ordered based on their absolute magnitude. The dissociation of AR from Heat Shock Proteins (HSP), components of the Akt signaling axis and MAPK activation were important (top 2% of sensitive interactions) irrespective of androgen status. Sequestered AR was unable to become activated by androgens or MAPK. Thus, increased AR-HSP dissociation promoted increased AR activation and AR-driven gene expression. Several components of the MAPK cascade were also important including Ras binding to GAP and Raf, and the dephosphorylation of ERK. The sensitivity of MAPK was not unexpected. ERK was critical to outlaw activation of AR. Moreover, ERK activation was modeled as being Ras dependent. We also found the Akt signaling axis to have components in the top 2% of sensitive interactions irrespective of androgen status. For example, the formation of PIP3, an early step in the PI3K/Akt signaling axis regulated by PTEN, was found to be highly sensitive in all clones. Looking beyond the upper 2% of sensitive interactions, additional common mechanisms were identified. These included AR interactions with DHT, recruitment of adapter molecules by Her2, activation of ERK by MEKpp and additional regulation of PIP3 formation by PTEN.

Translation interactions became more fragile while transcription became more robust with increasing androgen independence. Her2 auto-activation and Her2 cPAcP interactions were also increasingly important with increasing androgen independence. The difference in the importance of interactions in AI versus AD LNCaP clones was estimated by computing shifts in the sensitivity rankings (Table S5). In addition to considering C-33 and C-81, we analyzed a third clone, C-51, which was moderately androgen dependent. There were 117 statistically significant shifts (52 more and 65 less sensitive) between the C-81 and C-33 clones. However, only 14 shifts were larger than one standard above the mean shift. Of the 14 large shifts, 50% involved PSA and PAcP translation while the remainder were associated with Her2 and cPAcP. Conversely, PSA transcription became more robust with increasing androgen independence. Similarly, when comparing C-33 to C-51, PSA translation and Her2 activity became more sensitive with increasing androgen independence. Inspection of the importance of the final step in PSA transcription and translation among the individual models in the ensemble showed a shift away from transcription (Fig. 7C) toward translation (Fig. 7D) across the population of models. The increasing importance of translation was not limited to PSA, although PSA was the most significant example. Globally, 16 of the 52 interactions that were more sensitive in C-81 involved translation while only 4 of 52 involved transcription. No translation mechanisms became more robust in C-81 compared to C-33. Similar to PSA, translation of other key proteins such as cPAcP became more sensitive in C-81 versus C-33. Of the statistically significant shifts, 7/9 of the cPAcP translation interactions were more sensitive in C-81. Additionally, both mechanisms for the phosphorylation of 4E-BP1 by TOR kinase, a key step in translation initiation that liberates eIF4E, were also more importance in C-81. Taken together, the sensitivity analysis suggested that the fragility of the translational subsystem directly correlated to androgen independence.

**Figure 8:**
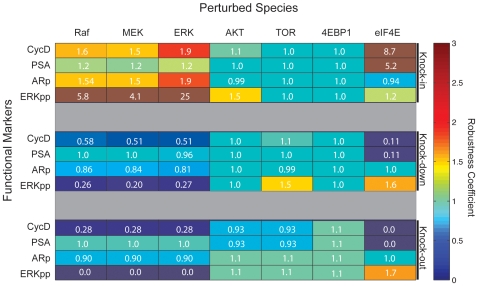
Robustness analysis of functional protein markers. The expression level of seven key proteins was altered by a factor of 10, .1 or 0 (knock-in, knock-down or knock-out) and robustness coefficients (area under the curve for the perturbed versus wild-type simulation) were calculated for cyclin D and PSA expression levels along with ERK and AR activation levels. Simulations were run for C-81, with the indicated perturbation, to approximate steady-state and 10nM of DHT was added for 72 hours. Ensemble mean values are reported.

To quantify the effects of perturbing key species in C-81 clones we preformed robustness analysis on four functional protein markers. The initial conditions of seven key protein species were altered by a factor of 10, .1 or 0 for knock-in, knock-down or knock-out perturbations, respectively. We then calculated the effect of these perturbation on cyclin D and PSA expression levels along with ERK and AR activation levels. Perturbation of Raf, MEK or ERK had similar effects on the functional markers with ERK being the most notable (Fig. 8, lanes 1, 2 and 3). Trivially, ERK perturbations directly effected ERK activation levels. However, more importantly, ERK perturbations greatly effected cyclin D expression levels. ERK knock-ins approximately doubled cyclin D while ERK knock-outs reduced cyclin D to less then one third of wild-type levels. The functional markers were robust to perturbations in AKT and TOR with differing effects on ERK activity and slight decreases in expression levels upon AKT or TOR knock-out (Fig. 8, lanes 4 and 5). Furthermore, the translation initiation factor eIF4E demonstrated a limiting reagent behavior in the expression of both cyclin D and PSA while perturbations in 4E-BP1 had little effect (Fig 8, lanes 6 and 7). However, the 4E-BP1 results could be an artifact of artificially high background levels of eIF4E as no direct eIF4E measurements were included in the training data. Knock-in simulations of eIF4E demonstrated an 8.7 and 5.2 fold increase in cyclin D and PSA expression. Reduction of eIF4E resulted in a 89% loss of expression and, full knock-out simulations predicted a complete loss of cyclin D and PSA.

### The MAPK and Akt Pathways Synergistically Activated Cyclin D Expression

**Figure 9:**
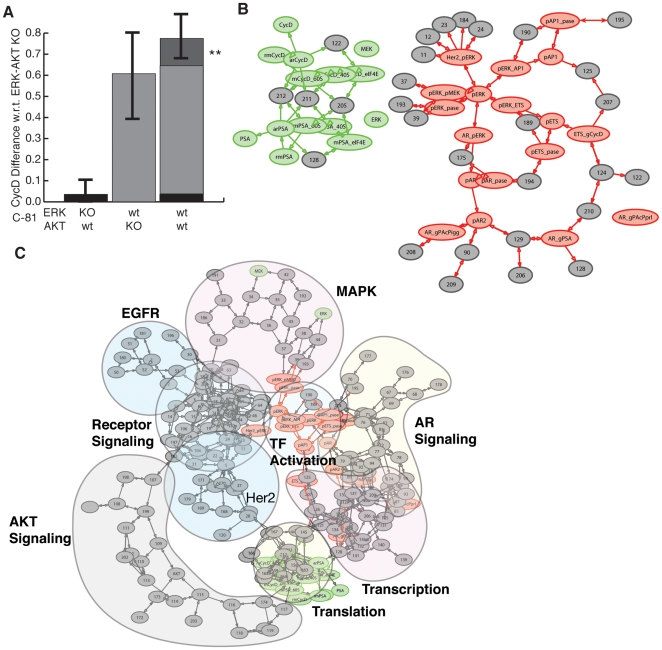
Synergy analysis between the ERK and Akt signaling axes in LNCaP C-81 cells. The double ERK and Akt knock-out was used as the control. **A**: The difference in steady state cyclin D expression (compared to the control) with the knock-in of Akt (left), ERK (center) and both (right). The predicted cyclin D levels were normalized by the basil C-81 steady state cyclin D level in each case. The error bars denote one standard deviation centered about the ensemble mean. The region denoted by the asterisks represents above-additive cyclin D expression. **B**: Species and interactions that demonstrated a positive (negative) synergy are shown as green (red) in the connectivity diagram. Species or interactions not effected are shown in grey. **C**: The full connectivity diagram qualitatively clustered in functional groups. Positive (negative) synergy are shown in green (red) in the connectivity diagram. Species or interactions not effected are shown in grey.

Complex systems composed of interacting subsystems can display emergent properties that are not explained by the individual subsystems alone [62]. In cancer biology, it is common to speak of signal transduction pathways as if they were isolated. In reality, these components are highly interconnected and can interact in a variety of ways sometimes leading to unpredictable behavior. In this study, we explored whether the MAPK and Akt signaling axes synergistically activated the expression of cyclin D. We compared the steady-state cyclin D expression in Akt and ERK knock-outs with wild-type C-81 cells in the absence of androgens. At steady-state, the MAPK and Akt pathways synergistically (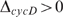) activated cyclin D expression in C-81 cells without androgen (Fig. 9A). Thus, steady-state cyclin D expression was *greater* in wild-type cells (Akt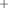-ERK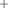) than the linear combination of cyclin D expression in Akt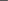-EKT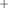 and Akt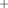-ERK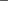 cells. The above-additive (superlinear) cyclin D expression was statistically significant within a 95% confidence interval. However, the relatively large standard deviation suggested that cyclin D expression varied widely across the ensemble. To address this, we inspected every model in the ensemble and found that each predicted an above-additive increase in cyclin D expression (data not shown). Superlinear cyclin D expression may be the result of positive synergy between the MAPK and translation subsystems. To elucidate the underlying mechanisms responsible for synergy we expanded the analysis to include all modeled species (both proteins and protein complexes) and rates. Many functional network subunits demonstrated no statistically significant deviations from additive behavior (Fig. 9C, grey). However, 22 species (79 interactions) were negatively coupled to Akt/ERK (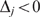; Fig. 9B, red) while 14 species (37 interactions) had a positive synergy (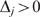; Fig. 9B, green). Synergy between the MAPK and Akt signaling subsystems negatively effected transcription factor activation. Phosphorylated ERK (ERKpp) activated AR (pAR), and the transcription factors AP1 and ETS all showed a below additive response (Fig. 9B). Conversely, positive synergy was almost exclusively limited to translation interactions. The binding of eIF4E, 40S and 60S ribosomes to form the mRNA initiation complex, elongation and termination steps all had positive synergy with ERK/Akt knockdowns (Fig. 9B).

## Discussion

A critical milestone in prostate cancer progression is the onset of androgen independence. In this study, we formulated and analyzed an ensemble of mathematical models of the androgen response of AI and AD LNCaP prostate cancer epithelial cells. The model ensemble was identified using 14 different steady-state and dynamic data sets taken from literature. With the exception of one study, all the training data was generated in LNCaP cell-lines. We estimated which molecular subsystems were important in AI versus AD cells using sensitivity analysis. For example, the assembly and regulation of Her2 adapter complexes and the regulation of ERK were sensitive irrespective of androgen status. The dissociation of AR from HSP was also in the top 2% of sensitive interactions for both C-33 and C-81. On the surface, the importance of AR in C-81 was surprising as the proliferation of C-81 is androgen independent. However, AR can be activated independently of androgen, thus, the presence of androgen is not required for androgen action [15], [63]. The differentiating factor between the AI and AD models described here was the expression rate of PAcP conformers. We demonstrated the ability of decreased PAcP expression to describe the PSA levels of increasingly androgen independent sub-lines. Moreover, interactions involving Her2 auto-phopshporylation, cPAcP availability and cPAcP phosphatase activity were significantly more fragile in C-81 versus C-33. These results suggest that the regulation of the phosphorylation state of Her2 by cPAcP may be a critical interaction controlling androgen action in the absence of hormone signals. Experimentally this has been demonstrated as forced expression of PAcP is sufficient to suppress C-81 xenograft tumor growth [64].

Model analysis suggested that translation interactions were more fragile and transcription more robust in AI versus AD cells. Globally, 16 of the 52 interactions that were more sensitive in C-81 involved translation while only 4 of 52 involved transcription. Moreover, no translation mechanisms became more robust in AI versus AD cells. The importance of translation in more aggressive cancers (increasing androgen independence) may be due, in-part, to synergies between the Akt and MAPK pathways. Simulations of ERK and/or Akt knockouts showed an above-additive response almost exclusively limited to translation upon the simultaneous reinstitution of Akt and ERK. *In-vivo* studies of AIPC have demonstrated positive synergies between the MAPK and Akt pathways. Gao *et al.* observed above-additive tumor growth rates in castrated and mock nude male mice upon the forced expression of constitutively active Akt and B-Raf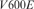 [65]. These experiments suggest that cell proliferation may be regulated by a complex integration of the MAPK and Akt signaling axes. Our robustness analysis suggested that independent perturbations in TOR and AKT may have little or no effect on AIPC. However, we observed the possibility of an inverse relationship between TOR and ERK activation. This suggests that if TOR or Akt were to be independently targeted, AKT might be a more suitable therapeutic target. Additionally, we observed that perturbations in Raf, MEK and ERK had a similar effect on cyclin D but not PSA expression, with ERK being more pronounced. Current therapeutics such as trastuzumab or gefitinib, which target either Her2 or EGFR respectively, have had little efficacy against hormone-refractory prostate cancers [66], [67]. Our results suggest that a possible factor in their lack of effectiveness is that they fail to address synergy between growth factor signaling, MAPK activation and the Akt signaling axes. Our analysis also demonstrated that translation mechanisms were generally more sensitive in increasingly androgen independent models. The translation results suggest that the direct targeting of the translation machinery may be useful for the treatment of AIPC. Our robustness analysis identified eIF4E as a limiting reagent in the expression of both cyclin D and PSA in C-81 clones. Soni *et al.* demonstrated the effectiveness of directly targeting eIF4E in breast cancer. Down-regulation of eIF4E resulted in decreased cyclin D expression and decreased growth rate without the deleterious effect of inhibitors such as rapamycin which act further upstream [68]. Previous modeling studies from our laboratory have also demonstrated the importance of translation beyond cyclin D [69]. However, the current model has only a basic description of translation initiation. Moreover, translation parameters were only indirectly trained from the PSA mRNA and protein data. Thus, while the initial robustness and sensitivity results are encouraging more studies are needed.

Analysis of the ensemble of AI models suggested the Akt and MAPK pathways synergistically enhanced cyclin D expression by up-regulating translation. Cyclin D is expressed early in the cell cycle and a point of convergence in the proliferative action of multiple receptors [70]. Many studies have identified a direct correlation between cyclin D regulation and prostate cancer, as well as breast and non-small cell lung cancer [71]–[73]. Balk *et al.* demonstrated that increased cyclin D expression in PTEN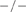 LNCaP cells following DHT addition was largely because of increased translation [74]. PTEN loss and presumably the activation of Akt has been implicated with increased translation and the resistance to therapeutics which target Her2 and EGFR [75], [76]. However, the underlying mechanism responsible for the increased translation in the Balk *et al.* study was not solely AKT dependent. Early translation activation was due to PI3K/Akt signaling but TOR activation at later time points was Akt independent. One key difference between the modeling and the Balk *et al.* study was the binding of activated AR with the regulatory subunit of PI3K. This interaction, which was not included in the model, was at least partially responsible for TOR activation and the eventual liberation of eIF4E. In addition to direct AR binding, PI3K (and subsequently TOR) can be activated through receptor adaptor complexes such as those associated with Her2. In the model, PI3K was activated by androgen (in the absence of growth factor) because of the down-regulation of cPAcP expression by activated AR. Upregulated PI3K then drove Akt dependent activation of TOR which led to enhanced liberation of eIF4E from 4E-BP1. Thus, while the initiating events driving TOR activation were different, the subsequent up-regulation of cyclin D translation was similar. This suggests that the model prediction of a complex synergy between interacting signaling axes may be valid. It also suggests a falsifiable hypothesis that cPAcP could be critical to enhanced translation following androgen stimulation.

The role of mechanistic mathematical modeling in drug design remains unclear. A common criticism of such techniques has been the poorly characterized effect of model uncertainty. Model uncertainty has two forms. Structural uncertainty is defined as uncertainty in the biology, while parametric uncertainty is defined as incomplete knowledge of parameter values. In this study, parametric uncertainty was minimized by considering a family of consistent models instead of a single best-fit but uncertain model. While model ensembles often poorly constrain individual parameter values, they may robustly constrain model predictions [56]. Structural uncertainty was addressed by considering only molecular interactions supported by experimental evidence. However, the current model contained some abstracted pathways and should be expanded to include additional biology. For example, the analysis highlighted the importance of translation. However, the current model contains a limited description of initiation factor activation and the assembly of the 80S initiation complex. A more detailed translation interaction network could further refine which translation components were important in AI versus AD cells. Another example is the mechanism by which AR transcriptionally regulates the expression of target genes. In the current model we ignored the role of transcriptional co-regulators and assumed activated AR functioned alone. While this is a reasonable first approximation, well known co-repressors and activators [77] such as ARA70 [78] should be included. The regulation and activity of these co-regulators may be different in AI versus AD cells and could enhance the list of differentially important targets. Additionally, a nuclear compartment and enhanced cell cycle and cell death subnetworks should be added to the model. These additional networks could be critical to understanding cell proliferation and survival effects in AI versus AD cells. For example, androgen and AR are known to regulate several components of the G1-phase of the cell cycle in prostate cells, not just cyclin D [79]. Moreover, the model describes the activation of Akt in the context of translation initiation, but not its well know survival functions [80], [81]. Lastly, given the importance of EGFR and Her2 induced MAPK activation in the current study and the therapeutic emphasis on receptor inhibition we plan to include a more complete receptor signaling network. Other receptors, IGFR and IL-6R have also been implicated in prostate cancer [82]–[84]. Understanding the signaling associated with these receptors and their downstream targets should be considered and will provide a better representation of how intra- extra-cellular communication drives cell fate decisions. Furthermore, the application of advanced sampling techniques may allow for a more exhaustive investigation of parameter space. For example, multi-objective optimization ensemble techniques could be used to balance conflicts in the training data [40]. Additionally, understanding the topological details of the cost function in an extended parameter space could provide statistical information on kinetic rates and initial conditions. Other techniques, for example the calculation of the mutual information matrix, could also provide insight into correlations between model interactions. Also, computation of second order sensitivity coefficients would allow the identification of possible synergies in the model. Thus, we expect that deeper insight could be generated by extending the network structure and through the application of advanced model analysis tools.

## Materials and Methods

### Formulation and Solution of the Model Equations

The prostate model was formulated as a set of coupled Ordinary Differential Equations (ODEs):
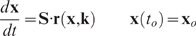
(1)The symbol 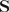 denotes the stoichiometric matrix (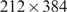). The quantity 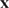 denotes the species concentration (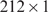). The term 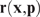 denotes the vector of reaction rates (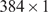). Each row in 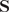 described a species while each column described the stoichiometry of network interactions. Thus, the 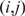 element of 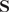, denoted by 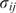, described how protein 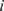 was involved in rate 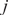. If 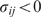, then protein 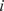 was consumed in 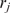. Conversely, if 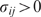, protein 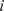 was produced by 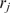. Lastly, if 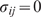, protein 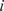 was not involved in rate 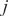.

We assumed mass-action kinetics for each interaction in the network. The rate expression for protein-protein interaction or catalytic reaction 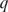:
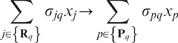
(2)was given by:
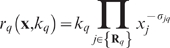
(3)The set 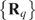 denotes reactants for reaction 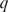. The quantity 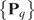 denotes the set of products for reaction 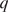. The 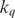 term denotes the rate constant governing the qth interaction. Lastly, 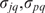 denote stoichiometric coefficients (elements of the matrix 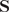). We treated every interaction in the model as non-negative. All reversible interactions were split into two irreversible steps. The mass-action formulation, while expanding the dimension of the prostate model, regularized the mathematical structure. The regular structure allowed automatic generation of the model equations. In addition, an analytical Jacobian (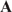) and matrix of partial derivatives of the mass balances with respect to the model parameters (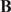) were also generated. Mass-action kinetics also regularized the model parameters. Unknown model parameters were one of only three types, association, dissociation or catalytic rate constants. Thus, although mass-action kinetics increased the number of parameters and species, they reduced the complexity of model analysis. In this study, we did not consider intracellular concentration gradients. However, we accounted for membrane and cytosolic proteins by explicitly incorporating separate membrane and cytosolic protein species. We did not consider a separate nuclear compartment.

### Simulation Protocol

An approximate steady-state was used as the starting point (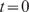 hr) for all simulations presented in this study. For example, when calculating the response of LNCaP to the addition of DHT, we first ran the model to steady-state and then simulated the addition of DHT. Although no individual cell is likely to be at steady-state we assumed that steady-state was a reasonable approximation of the population average behavior of LNCaP cells growing in the exponential phase. The steady-state was estimated numerically by repeatedly solving the model equations and estimating the difference between two subsequent time points:
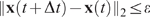
(4)The quantities 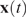 and 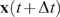 denote the simulated concentration vector at time 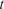 and 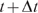, respectively. The quantity 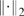 denotes the 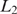 vector norm. In this study, we used 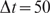 hrs of simulated time and 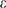 = 0.01 for all simulations.

### Estimation of the Prostate Model Parameter Ensemble

An initial set of model parameters, denoted by 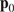, was chosen by hand to replicate the training data. The training data consisted of 14 time-series and steady-state data sets taken from literature sources (Table S2). The initial parameter guess 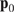 was used to generate an ensemble of parameters that maximized the likelihood of describing the training data. The difference between the measured and simulated value of species 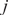 at time or condition 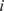, denoted by 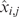 and 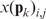 respectively, was quantified by the normalized mean squared error, 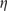:
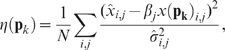
(5)where the sum was carried out over all species 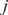 and observations 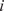. The quantities 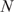 and 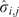 denote the total number of observations and the measurement standard deviation of species 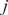 at time or condition 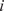, respectively. If no experimental error was reported, we assumed a standard deviation equal to 10% of the reported observation. In cases where the quantification of the stimulus or observation was unclear an augmented error of 20%–100% was applied to compensate for the added uncertainty. 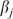 is a scaling factor which is required when considering experimental data that is accurate only to a multiplicative constant (assumed here to be the case form immunobloting analysis). The scaling factor was chosen to minimize the normalized squared error between a given experiment and species 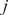 [54]:
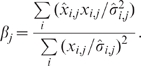
(6)Because of the scaling factor, the concentration units on simulation results were arbitrary (consistent with the arbitrary units on the majority of the training data). All simulation outputs reported in this study were scaled by the corresponding 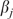. There was insufficient training data to properly constrain the 420 model parameters. To account for parametric uncertainty, a monte-carlo approach similar to Battogtokh *et al.* [52] was used to generate an ensemble of parameters. Consider a set of model parameters 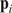. Let the likelihood that model simulations with parameters 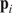 describe the training data be defined as:
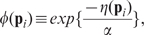
(7)where 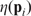 denotes the simulation error associated with parameter set 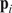. The quantity 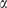 is a parameter used to tune the rate of acceptance. Further let the acceptance probability, 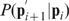, of a new parameter set, 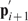, be 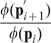 if 
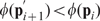
 and 1 otherwise. 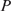 denotes the probability that 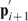 will be accepted as 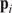 for consecutive monte-carlo steps. Parameter sets were generated by applying a small additive random perturbation in log space:
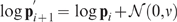
(8)where 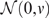 is a normally distributed random number with zero mean and variance 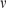. The perturbation was applied in log space to account for the large variation in parameter scales and to ensure positivity. Monte-carlo trajectories were generated starting from 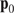 where 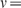
0.05 or 0.1 and 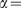
1 or 0.5. The autocorrelation function of each trajectory was calculated. The number of monte-carlo steps between parameter sets which were added to the ensemble was taken to be the number of steps after which the autocorrelation function dropped to 5% of its initial value. This was done to ensure independence between sets in the ensemble. To compensate for noise in the autocorrelation function an exponential fit was applied. The final ensemble contained 107 parameter sets, which produced an ensemble 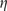 of 5.25.

### Sensitivity Analysis of the Prostate Network

Overall State Sensitivity Coefficients (OSSC) were used to estimate which structural elements of the prostate network were sensitive [35]. OSSC values were determined by first calculating the first-order sensitivity coefficients at time 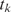:
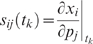
(9)First-order sensitivity coefficients were computed by solving the matrix differential equation:
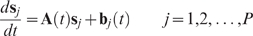
(10)subject to the initial condition 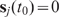. In Eqn. 10, 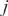 denotes the parameter index, 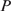 denotes the number of parameters in the model, 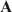 denotes the Jacobian matrix, and 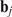 denotes the 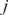
th column of the matrix of first-derivatives of the mass balances with respect to the parameter values (denoted by 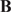). An analytical Jacobian and matrix of first-derivatives of the mass balances w.r.t the parameters:
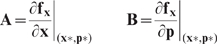
(11)were generated from the model equations. The quantity 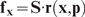 and 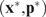 denotes a point along the unperturbed model solution. The sensitivity equations required that we solve the model equations to evaluate the 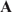 and 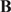 matrices. Thus, we formulated the sensitivity problem as an extended kinetic-sensitivity system of equations [85]:
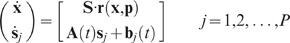
(12)where 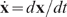 and 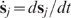. We solved the kinetic-sensitivity system for multiple parameters in a single calculation using the LSODE routine of OCTAVE (www.octave.org). The first-order sensitivity coefficients were then used to calculate the OSSC value for parameter 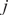:
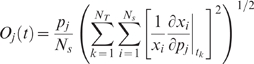
(13)The terms 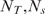 denote the number of time points considered and the state dimension of the model, respectively. To account for parametric uncertainty, OSSC values were calculated over the parameter ensemble. Parameters were ranked-ordered (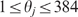) based upon the magnitude of the OSSC value. Large values of 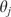 indicated fragile or important interactions in the prostate network architecture. Conversely, small values of 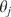 indicated robustness.

Each model in the ensemble was run to approximately steady state. At steady-state, 10nM DHT was added and the first order sensitivity coefficients were calculated for 100 seconds of simulated time. OSSC values were then calculated and the rank ordering determined. We collected interactions whose rank was at least one standard deviation above the mean rank calculated over all parameters. Highly ranked interactions were statistically significantly different between LNCaP clones if the null hypothesis could be rejected with 95% confidence via a t-test. To estimate significance, we performed a two variable unequal variance double tail t-test using the MATLAB (R) statistical toolbox (2007a, The Mathworks, Natick, MA).

### Robustness Analysis of Functional Protein Markers

Robustness coefficients of the form:
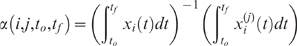
(14)were calculated to understand the regulatory connectedness of functional protein markers in the LNCaP network. The robustness coefficient 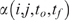 is the ratio of the integrated concentration of a network output in the presence (numerator) and absence (denominator) of structural or operational perturbation. Here 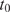 and 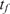 denote the initial and final simulation time respectively. Simulations were taken of C-81 from approximate steady-state at 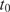, 10nM of DHT was added at 1 hour and 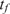 was taken to be 72 hours after DHT addition. The network output was taken to be the network states. The quantity 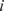 denotes the index for a marker or reference species while 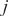 denotes the perturbation index, respectively. If 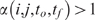, then the perturbation *increases* the output concentration. Conversely, if 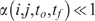 the perturbation *decreases* the output concentration. Lastly, if 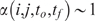 the perturbation does not influence the output concentration.

### Calculation of Steady-State Synergy Coefficients

To understand the connectedness of subsystems in the prostate network following ERK and/or Akt knockdowns we computed synergy coefficients of the form:
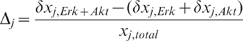
(15)The quantity 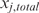 denotes the steady-state concentration (flux) of species (interaction) j in wild-type C-81. The quantity 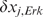 (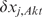) denotes the steady-state concentration (flux) of species (interaction) j in the presence of an Akt (ERK) knock-out minus the basal value of quantity j. The term 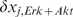 denotes the steady-state concentration (flux) of species (interaction) j in wild-type C-81. If 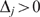, the quantity j has a positive synergy with Akt and ERK. In other words, the steady-state concentration (flux) of species (interaction) j in the wild-type was *greater* than the sum of the individual contributions in single Akt or ERK knock-downouts. Conversely, if 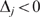, the quantity j has a negative synergy with Akt and ERK. Lastly, if 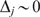 then there is no connection between quantity j and the Akt/ERK signaling axes.

## Supporting Information

Table S1 Prostate model interactions and parameters for the C-33, C-51, and C-81 LNCaP clones. The kinetics of binding and catalytic interactions were assumed to follow mass-action rate laws. The quantity kon denotes forward rate constants, koff denotes backward rate constants, and kcat denotes catalytic rate constants. All binding interactions were assumed to be reversible. The citations listed were the primary source of information for the corresponding interaction, and include either the exact interaction (i.e., from preexisting model) or evidence from which the interaction was inferred. Unless otherwise specified, concentration units were arbitrary (A.U) as a result of arbitrary units on training data. Thus, zero-order rate constants had units of A∶Us¡1, first-order rate constants had units of s¡1, and second-order rate constants had units of (A∶U)¡1s¡1 The mean and standard deviation over the parameter ensemble are reported for each kinetic parameter. ¦: The expression of the PAcP isoforms, PSA, and cyclin D was implemented using the same translation/transcription heuristic, save any specific transcription factors. ?: Her2 adaptor complex reactions were taken to be similar those of EGFR (66). y: Inferred from collaboration with Prosetta Cooperation (http://www.prosetta.com/). z: Internalized EGFR complexes were assumed to signal identically to membrane-bound EGFR (30,67).
(0.07 MB XLS)

Table S2 Experimental training data used to estimate the ensemble of prostate model parameters.
(0.02 MB PDF)

Table S3 Non-zero initial conditions estimated from the training data for the C-33 LNCaP clone. The mean (μ) and standard deviation (σ) calculated over the ensemble are shown.
(0.03 MB PDF)

Table S4 Interactions determined to be significantly fragile for the C-33, C-51, and C-81 LNCaP clones. Overall state sensitivity coefficients (OSSCs) were calculated over the parameter ensemble. The OSSC values were ranked ordered. The mean rank and standard deviation for interactions with rank greater than at least one standard deviation above the overall mean rank are reported.
(0.03 MB PDF)

Table S5 Statistically significant sensitivity differences between AI and AD LNCaP clones. Negative changes in the mean rank denote interactions that were more sensitive in AI versus AD cells.
(0.02 MB PDF)
