# Supplementary material for: Analysis of the Molecular Networks in Androgen Dependent and Independent Prostate Cancer Revealed Fragile and Robust Subsystems
Source: PLoS One. 2010 Jan 28;5(1):e8864. doi: 10.1371/journal.pone.0008864 (PMC2812491; doi:10.1371/journal.pone.0008864)
Supplement: Table S2 — Experimental training data used to estimate the ensemble of prostate model parameters. (0.02 MB PDF) [file pone.0008864.s002.pdf]

Table S2: Experimental training data used to estimate the ensemble of prostate model parameters.

Experimental Setup	Observation	citation	use
C-81 transfection with WT PAcP	cyclin D 0.7 of C-81 WT	†	training
C-81 transfection with cPAcP	cyclin D 0.3 of C-81 WT	†	training
C-81 transfection with sPAcP	cyclin D 1.1 fold of WT C-81	†	training
Addition of 10nm DHT to C-81	PSA increased over time	(14)	training
Addition of 10nM DHT to C-33	PSA & Her2 activation increased and PAcP decreased	(14)	training
Overexpression of Her2 in C-33	PSA mRNA 2.2 fold of C-33 WT	(18)	training
Overexpression of Her2 in C-33	PSA 2 fold of C-33 WT	(14)	training
Transfection of constitutively active MEK in C-33	PSA 3 fold of C-33 WT	(14)	training
1μM MEK inhibitor PD98059 in C-81	PSA 0.6 of C-81 WT	(14)	training
MEK inhibitor PD98059 in C-33 with Her2 overexpression	PSA 1.86 of C-33 WT (compared to 2 with Her2 overexpression alone)	(14)	training
0.5μM Her2 inhibitor AG879 in C-81	PSA 0.58 of C-81 WT	(14)	training
10μM Her2 inhibitor AG879 in C-81	PSA 0.17 of C-81 WT	(14)	training
EGFR inhibitor AG1478 in C-81	no PSA effect	(14)	training
50ng/ml EGF to HeLa cell culture	transient increase in Erk activity	(30)	training
0.1nM DHT added to AD LNCaP	increased cyclin D expression	(57)	validation
2nM milbolerone added to AD LNCaP	Increased PSA secretion	(58)	validation
2nM milbolerone added to AD LNCaP with antisense AR	Increased PSA secretion to lower extent then WT	(58)	validation
