# Supplementary material for: Analysis of the Molecular Networks in Androgen Dependent and Independent Prostate Cancer Revealed Fragile and Robust Subsystems
Source: PLoS One. 2010 Jan 28;5(1):e8864. doi: 10.1371/journal.pone.0008864 (PMC2812491; doi:10.1371/journal.pone.0008864)
Supplement: Table S3 — Non-zero initial conditions estimated from the training data for the C-33 LNCaP clone. The mean (μ) and standard deviation (σ) calculated over the ensemble are shown. (0.03 MB PDF) [file pone.0008864.s003.pdf]

Table S3: Non-zero initial conditions estimated from the training data for the C-33 LNCaP clone. The mean (μ) and standard deviation (σ) calculated over the ensemble are shown.

species	initial condition ($\mu \pm \sigma$)
Pase7	24.71 $\pm$ 23.66
AR	192.40 $\pm$ 260.66
HSP	486.15 $\pm$ 659.55
Rase5a	81.11 $\pm$ 66.98
Her2	131.57 $\pm$ 111.41
EGFR	115.41 $\pm$ 82.19
Shc	91.19 $\pm$ 71.89
Grb2	80.32 $\pm$ 67.10
Sos	35.79 $\pm$ 29.37
Ras-GDP	233.39 $\pm$ 456.75
Raf	76.83 $\pm$ 54.31
MEK	1572.31 $\pm$ 2260.09
ERK	587.24 $\pm$ 401.24
ETS	133.52 $\pm$ 150.03
AP1	107.34 $\pm$ 172.51
Pase1	181.67 $\pm$ 513.24
Pase2	20.88 $\pm$ 11.78
Pase3	22.76 $\pm$ 12.44
Pase5	65.13 $\pm$ 82.13
Pase6	168.28 $\pm$ 234.48
GAP	60.71 $\pm$ 100.62
PI3K	174.54 $\pm$ 240.45
PtdIns2	131.27 $\pm$ 122.14
PtdIns3	119.05 $\pm$ 94.11
PTEN	123.84 $\pm$ 142.99
Akt	332.36 $\pm$ 585.30
Pdk1	190.88 $\pm$ 237.37
TOR	121.80 $\pm$ 120.53
4E-BP1	136.67 $\pm$ 107.57
eIF4E	3707.42 $\pm$ 3178.77
g-PSA	3.29 $\pm$ 1.87
g-CycD	2.90 $\pm$ 4.98
g-cPAcP	0.09 $\pm$ 0.06
g-sPAcP	0.11 $\pm$ 0.09

continued on next page

continued from previous page

species	initial condition ($\mu \pm \sigma$)
RNAp	371.62 $\pm$ 314.59
40S	8203.19 $\pm$ 16956.30
60S	4732.73 $\pm$ 4700.66
