# Supplementary material for: Analysis of the Molecular Networks in Androgen Dependent and Independent Prostate Cancer Revealed Fragile and Robust Subsystems
Source: PLoS One. 2010 Jan 28;5(1):e8864. doi: 10.1371/journal.pone.0008864 (PMC2812491; doi:10.1371/journal.pone.0008864)
Supplement: Table S4 — Interactions determined to be significantly fragile for the C-33, C-51, and C-81 LNCaP clones. Overall state sensitivity coefficients (OSSCs) were calculated over the parameter ensemble. The OSSC values were ranked ordered. The mean rank and standard deviation for interactions with rank greater than at least one standard deviation above the overall mean rank are reported. (0.03 MB PDF) [file pone.0008864.s004.pdf]

Table S4: Interactions determined to be significantly fragile for the C-33, C-51 and C-81 LNCaP clones. Overall State Sensitivity Coefficients (OSSCs) were calculated over the parameter ensemble. The OSSC values were ranked ordered. The mean rank and standard deviation for interactions with rank greater than at least one standard deviation above the overall mean rank are reported.

Reaction	mean rank	st. dev rank	p-value
**C-33**			
AR-p-DHT-2+g-sPAcP→AR-p-DHT-2-g-sPAcP	302.4059406	15.97843734	0.039916369
Ras-GTP-Raf→Ras-GTP+Raf-p	302.7821782	18.9159705	0.046713871
AP1-p-Pase6→AP1+Pase6	302.8118812	9.99773526	0.00082649
ETS-p+Pase5→ETS-p-Pase5	303.0693069	17.82547816	0.02648256
mRNA-sPAcP-eIF4E+40S→mRNA-sPAcP-eIF4E-40S	303.4851485	11.65648068	0.000567415
Pdk1-m+Akt-m→Pdk1-m-Akt-m	303.960396	8.723384746	1.11E-06
ETS-p-Pase5→ETS+Pase5	304.3465347	12.61418002	0.000133415
AP1-p+Pase6→AP1-p-Pase6	309.0891089	31.45076272	0.001584816
mRNA-sPAcP-eIF4E-40S-60S→Rm-sPAcP+eIF4E	309.8118812	21.69943781	3.90E-06
2*AR-p-DHT→AR-p-DHT-2	313.5643564	27.07211339	6.11E-07
mRNA-sPAcP→[]	315.7524752	63.3152424	0.006070198
2*Her2-2-p+cPAcP-2→2Her2-2-p-cPAcP-2	315.8613861	33.36003113	2.00E-06
ERK-pp-Pase3→ERK-p+Pase3	318.5148515	49.25034329	0.000106094
mRNA-CycD-eIF4E-40S+60S→mRNA-CycD-eIF4E-40S-60S	318.8019802	31.76024659	1.25E-08
mRNA-CycD-eIF4E+40S→mRNA-CycD-eIF4E-40S	318.9306931	33.70966822	5.07E-08
AR-p-DHT+Pase5→AR-p-DHT-Pase5	319.3663366	26.60556138	1.80E-11
Her2-2-sPAcP→Her2-2-p+sPAcP	321.8217822	25.22037368	1.90E-14
Raf-p-Pase1→Raf+Pase1	323.7821782	22.38130583	8.18E-19
Act-Akt-Pase7→Akt+Pase7	325.4158416	60.54411162	2.25E-05
Her2-2-p-Shc-p-Grb2-Sos+ERK-pp→Her2-2-p-Shc-p-Grb2-Sos-ERK-pp	326.8811881	33.90589397	8.76E-13
g-PSA-AR-p-DHT-2-RNAp→g-PSA-AR-p-DHT-2+RNAp+mRNA-PSA	327.990099	32.83064707	4.36E-14
g-PSA+AR-p-DHT-AR-p→g-PSA-AR-p-DHT-AR-p	328.1584158	15.42386775	2.07E-34
g-cPAcP-RNAp→g-cPAcP+RNAp	329.8217822	23.27042743	1.65E-23
AR-DHT→AR-p-DHT	330.039604	16.97809934	2.49E-33
ERK-pp-ETS→ERK-pp+ETS-p	330.4356436	22.63241163	7.61E-25
Her2-2-p-Shc-p-Grb2-Sos+Ras-GDP→Her2-2-p-Shc-p-Grb2-Sos-Ras-GDP	330.7425743	29.10705861	1.40E-18
Shc-p→Shc	331.5346535	14.6216741	3.60E-40
Her2-2-p+cPAcP→Her2-2-p-cPAcP	331.5346535	28.40846491	9.25E-20
ERK-pp+Pase3→ERK-pp-Pase3	332.1980198	31.79669944	1.34E-17
mRNA-cPAcP-eIF4E-40S+60S→mRNA-cPAcP-eIF4E-40S-60S	332.2871287	39.08921926	1.83E-13

continued on next page

continued from previous page

Reaction	mean rank	st. dev rank	p-value
AR+DHT→AR-DHT	335.4950495	28.38861289	8.88E-23
Ras-GTP-GAP→Ras-GTP+GAP	335.5049505	50.04604291	6.32E-11
ERK-pp-AP1→ERK-pp+AP1-p	336.960396	15.82823695	5.38E-43
mRNA-cPAcP-eIF4E+40S→mRNA-cPAcP-eIF4E-40S	336.980198	41.72896522	9.27E-15
Her2-2-p-Shc-p-Grb2+Sos→Her2-2-p-Shc-p-Grb2-Sos	338.0990099	35.12960585	3.83E-19
MEK-pp-Pase2→MEK-pp+Pase2	338.2871287	39.07173819	5.55E-17
ERK-pp-AP1→ERK-pp+AP1	338.7029703	18.00524926	5.81E-40
Her2-2+sPAcP→Her2-2-sPAcP	338.8415842	29.06930356	1.40E-24
ERK-pp-ETS→ERK-pp+ETS	339.5148515	23.01511078	3.15E-32
MEK-pp+Pase2→MEK-pp-Pase2	339.7227723	38.86613706	9.42E-18
mRNA-cPAcP+eIF4E→mRNA-cPAcP-eIF4E	339.8910891	35.54133028	5.79E-20
PtdIns3+PTEN→PtdIns3-PTEN	340.039604	27.87700028	1.17E-26
Grb2+Sos→Grb2-Sos	341.3564356	37.79192228	2.44E-19
Her2-2-p-Shc-p-Grb2→Her2-2-p-Shc-p+Grb2	341.3861386	30.78477517	9.83E-25
Her2-2-p-Grb2→Her2-2-p+Grb2	341.5742574	21.43160733	2.56E-36
ERK-MEK-pp→ERK-p+MEK-pp	342.2673267	31.13461386	5.26E-25
MEK-p+Raf-p→MEK-p-Raf-p	343.5643564	37.26023438	5.78E-21
g-sPAcP+RNAp→g-sPAcP-RNAp	343.960396	21.50013622	3.89E-38
ERK-pp+AP1→ERK-pp-AP1	344.7227723	12.55571253	2.64E-59
AR-p-DHT+AR-p→AR-p-DHT-AR-p	345.8217822	17.42957316	2.33E-47
mRNA-sPAcP+eIF4E→mRNA-sPAcP-eIF4E	346.6435644	34.26818637	4.71E-25
ERK-p+Pase3→ERK-p-Pase3	346.9009901	24.64179214	1.26E-35
ERK-p+MEK-pp→ERK-p-MEK-pp	347.3465347	27.47147419	2.69E-32
sPAcP→sPAcP-e	348.2673267	27.44351213	5.87E-33
Her2-2-p-Shc-p+Grb2→Her2-2-p-Shc-p-Grb2	348.5346535	21.65689443	2.17E-41
g-PSA+AR-p-DHT-2→g-PSA-AR-p-DHT-2	348.7623762	17.48331511	1.32E-49
ERK-pp+ETS→ERK-pp-ETS	349.5346535	11.96328075	2.12E-65
g-cPAcP+RNAp→g-cPAcP-RNAp	349.8316832	6.127473123	7.84E-94
PtdIns3-PTEN→PtdIns2+PTEN	350.0990099	29.71098572	1.44E-31
g-sPAcP-RNAp→g-sPAcP+RNAp+mRNA-sPAcP	352.4851485	40.24617659	8.00E-24
Ras-GTP-GAP→Ras-GDP+GAP	353.2376238	19.0482184	1.17E-49
Her2-2-p-Grb2-Sos+ERK-pp→Her2-2-p-Grb2-Sos-ERK-pp	354.019802	21.93102608	7.57E-45
Her2-2-p-Shc-p→Her2-2-p+Shc-p	355.3168317	18.11565195	4.27E-53
Her2-2-p-Grb2-Sos+Ras-GDP→Her2-2-p-Grb2-Sos-Ras-GDP	355.4356436	18.33754782	1.06E-52
Her2-2-p+Grb2-Sos→Her2-2-p-Grb2-Sos	361.0792079	35.52873141	3.78E-32
Raf-p+Pase1→Raf-p-Pase1	361.6237624	16.52651724	4.71E-61
Her2-2-p+Shc→Her2-2-p-Shc	363.1485149	16.06569812	3.41E-63
Ras-GTP+Raf→Ras-GTP-Raf	366.8712871	9.359342626	3.28E-88
PtdIns2-Act-PI3K→PtdIns3+Act-PI3K	367.6732673	19.44024045	2.98E-58

continued on next page

continued from previous page

Reaction	mean rank	st. dev rank	p-value
Ras-GTP+GAP→Ras-GTP-GAP	368.3861386	6.688204923	1.46E-103
AR-HSP→AR+HSP	375.4950495	17.27851282	1.70E-67
ERK-p-Pase3→ERK+Pase3	377.1584158	15.50453953	6.74E-73
ERK+MEK-pp→ERK-MEK-pp	381.029703	4.115788113	7.95E-132
**C-51**			
AP1-p-Pase6→AP1+Pase6	302.1578947	8.575750131	0.001745756
Ras-GTP-Raf→Ras-GTP+Raf-p	303	19.88599084	0.045952102
ETS-p+Pase5→ETS-p-Pase5	303.3157895	16.59502979	0.014236152
Pdk1-m+Akt-m→Pdk1-m-Akt-m	303.7368421	9.106632084	9.61E-06
ETS-p-Pase5→ETS+Pase5	303.9473684	11.5297039	0.000161664
mRNA-sPAcP-eIF4E+40S→mRNA-sPAcP-eIF4E-40S	304.9684211	15.17439161	0.000369539
Her2-2-p-cPAcP→Her2-2+cPAcP	308.5578947	24.48144981	0.000269085
AP1-p+Pase6→AP1-p-Pase6	310.2421053	25.34383172	4.28E-05
mRNA-sPAcP-eIF4E-40S-60S→Rm-sPAcP+eIF4E	313.2947368	22.27666879	1.83E-08
mRNA-CycD-eIF4E-40S+60S→mRNA-CycD-eIF4E-40S-60S	313.7052632	33.07913118	3.50E-05
g-PSA-AR-p-DHT-2-RNAp→g-PSA-AR-p-DHT-2+RNAp+mRNA-PSA	313.8421053	38.17282467	0.000222221
2*AR-p-DHT→AR-p-DHT-2	314.1263158	24.0220259	2.85E-08
ERK-pp-Pase3→ERK-p+Pase3	316.9157895	48.00365406	0.000338143
mRNA-sPAcP→[]	317.5684211	63.22635237	0.003381905
2*Her2-2-p+cPAcP-2→2Her2-2-p-cPAcP-2	317.8421053	33.02608723	2.69E-07
AR-p-DHT+Pase5→AR-p-DHT-Pase5	318.0736842	24.49156336	3.60E-11
mRNA-CycD-eIF4E+40S→mRNA-CycD-eIF4E-40S	318.5894737	34.67548648	3.28E-07
Raf-p-Pase1→Raf+Pase1	321.3578947	22.06922878	6.66E-16
Act-Akt-Pase7→Akt+Pase7	325.3684211	60.78799269	4.01E-05
Her2-2-sPAcP→Her2-2-p+sPAcP	326.5789474	27.72708307	1.28E-15
ERK-pp+Pase3→ERK-pp-Pase3	326.6526316	33.45374447	3.03E-12
Her2-2-p-Shc-p-Grb2-Sos+ERK-pp→Her2-2-p-Shc-p-Grb2-Sos-ERK-pp	327.9473684	37.42933508	3.32E-11
g-PSA+AR-p-DHT-AR-p→g-PSA-AR-p-DHT-AR-p	328.4842105	14.39799925	4.01E-35
AR-DHT→AR-p-DHT	329.1052632	16.30271123	8.22E-32
g-cPAcP-RNAp→g-cPAcP+RNAp	329.2736842	23.57449206	1.81E-21
Her2-2-p-Shc-p-Grb2-Sos+Ras-GDP→Her2-2-p-Shc-p-Grb2-Sos-Ras-GDP	331.3368421	29.15859015	5.32E-18
mRNA-cPAcP-eIF4E-40S+60S→mRNA-cPAcP-eIF4E-40S-60S	331.4	39.66926425	4.29E-12
ERK-pp-ETS→ERK-pp+ETS-p	331.8	19.2636008	2.36E-29
Ras-GTP-GAP→Ras-GTP+GAP	333.0842105	51.16406287	3.58E-09
Shc-p→Shc	333.3684211	15.7600779	2.70E-37
ERK-pp-AP1→ERK-pp+AP1	336.7263158	20.51158446	8.55E-32
ERK-pp-AP1→ERK-pp+AP1-p	337.0736842	12.72150447	1.64E-48
MEK-pp-Pase2→MEK-pp+Pase2	337.2947368	37.76265872	3.96E-16

continued on next page

continued from previous page

Reaction	mean rank	st. dev rank	p-value
mRNA-cPAcP-eIF4E+40S→mRNA-cPAcP-eIF4E-40S	337.3157895	42.40786004	7.00E-14
ERK-pp-ETS→ERK-pp+ETS	337.3473684	23.58267358	4.79E-28
AR+DHT→AR-DHT	338.2	24.96844324	5.00E-27
MEK-pp+Pase2→MEK-pp-Pase2	338.7157895	37.64590232	5.70E-17
Her2-2-p-Shc-p-Grb2+Sos→Her2-2-p-Shc-p-Grb2-Sos	338.8105263	34.82597178	9.32E-19
Grb2+Sos→Grb2-Sos	338.9052632	39.71532216	6.11E-16
PtdIns3+PTEN→PtdIns3-PTEN	339.5684211	29.20838114	1.27E-23
Her2-2-p+cPAcP→Her2-2-p-cPAcP	340.0947368	29.00637813	3.67E-24
ERK-p+Pase3→ERK-p-Pase3	341.2842105	26.78949643	3.28E-27
Her2-2+sPAcP→Her2-2-sPAcP	342.4421053	30.45193479	2.28E-24
ERK-pp+AP1→ERK-pp-AP1	342.5894737	16.21597311	1.12E-44
Her2-2-p-Shc-p-Grb2→Her2-2-p-Shc-p+Grb2	342.7684211	25.81287016	2.40E-29
Her2-2-p-Grb2→Her2-2-p+Grb2	342.7789474	21.45407774	3.41E-35
g-sPAcP+RNAp→g-sPAcP-RNAp	342.9894737	21.4478534	2.31E-35
MEK-p+Raf-p→MEK-p-Raf-p	343.1578947	38.42622892	6.58E-19
ERK-MEK-pp→ERK-p+MEK-pp	343.7473684	27.89075245	1.05E-27
ERK-p+MEK-pp→ERK-p-MEK-pp	344.2315789	27.88791575	4.97E-28
mRNA-cPAcP+eIF4E→mRNA-cPAcP-eIF4E	345.6842105	36.12296635	8.80E-22
Her2-2-p-Shc-p+Grb2→Her2-2-p-Shc-p-Grb2	346.4315789	23.27666671	3.54E-35
AR-p-DHT+AR-p→AR-p-DHT-AR-p	346.8315789	16.13929669	2.98E-48
g-PSA+AR-p-DHT-2→g-PSA-AR-p-DHT-2	347.2315789	16.73725918	3.13E-47
ERK-pp+ETS→ERK-pp-ETS	347.6736842	12.66611069	5.41E-58
mRNA-sPAcP+eIF4E→mRNA-sPAcP-eIF4E	347.7473684	35.32643812	1.66E-23
PtdIns3-PTEN→PtdIns2+PTEN	349.4105263	30.11608933	5.28E-29
g-cPAcP+RNAp→g-cPAcP-RNAp	349.7157895	6.308996347	4.17E-87
sPAcP→sPAcP-e	351.0105263	28.577317	1.40E-31
Ras-GTP-GAP→Ras-GDP+GAP	351.9473684	19.54340294	4.92E-45
g-sPAcP-RNAp→g-sPAcP+RNAp+mRNA-sPAcP	351.9578947	40.97365413	8.11E-22
Her2-2-p-Grb2-Sos+Ras-GDP→Her2-2-p-Grb2-Sos-Ras-GDP	352.1789474	20.4743634	1.57E-43
Her2-2-p-Shc-p→Her2-2-p+Shc-p	354.9894737	16.77372302	1.03E-52
Her2-2-p-Grb2-Sos+ERK-pp→Her2-2-p-Grb2-Sos-ERK-pp	355.8105263	19.06625276	1.64E-48
Her2-2-p+Grb2-Sos→Her2-2-p-Grb2-Sos	358.6631579	37.79657755	2.57E-27
Her2-2-p+Shc→Her2-2-p-Shc	361.2315789	19.83800321	1.95E-50
Raf-p+Pase1→Raf-p-Pase1	361.5157895	16.62646157	2.99E-57
Ras-GTP+Raf→Ras-GTP-Raf	366.5789474	9.591200954	6.39E-82
PtdIns2-Act-PI3K→PtdIns3+Act-PI3K	367.2210526	20.72790082	3.00E-52
Ras-GTP+GAP→Ras-GTP-GAP	367.8210526	7.643337584	8.85E-92
ERK-p-Pase3→ERK+Pase3	376.9894737	14.42986285	2.04E-71
AR-HSP→AR+HSP	377.8210526	5.201171826	6.56E-113

continued on next page

continued from previous page

Reaction	mean rank	st. dev rank	p-value
ERK+MEK-pp→ERK-MEK-pp	380.2315789	5.008314417	1.12E-115
**C-81**			
ETS-p-Pase5→ETS+Pase5	301.1604938	11.5297039	0.044372614
Pdk1-m+Akt-m→Pdk1-m-Akt-m	304.308642	9.106632084	6.31E-05
Her2-2-p-Shc-p-Grb2-Sos-ERK-pp→Her2-2-p-Shc-p-Grb2+Sos+ERK-pp	304.7160494	18.6392907	0.044183504
ETS-p+Pase5→ETS-p-Pase5	305.2222222	16.59502979	0.000259343
Rm-cPAcP→Ar-cPAcP	305.9259259	35.39776978	0.035058001
ERK-pp+Pase3→ERK-pp-Pase3	306.8765432	33.45374447	0.006750525
mRNA-sPAcP-eIF4E+40S→mRNA-sPAcP-eIF4E-40S	306.8888889	15.17439161	0.000275699
AP1-p+Pase6→AP1-p-Pase6	309.037037	25.34383172	8.85E-10
Her2-2-p-Shc→Her2-2-p-Shc-p	309.5925926	28.82266729	0.001615564
AR-p-DHT+Pase5→AR-p-DHT-Pase5	310.0246914	24.49156336	9.34E-05
ERK-p+Pase3→ERK-p-Pase3	311.1234568	26.78949643	0.000360203
Her2-2-p-Grb2-Sos-ERK-pp→Her2-2-p-Grb2+Sos+ERK-pp	313.0123457	25.30996979	0.000134248
mRNA-CycD-eIF4E+40S→mRNA-CycD-eIF4E-40S	314.0123457	34.67548648	3.02E-05
mRNA-sPAcP-eIF4E-40S-60S→Rm-sPAcP+eIF4E	314.1358025	22.27666879	2.25E-06
mRNA-sPAcP→[]	314.1604938	63.22635237	0.010774155
2*AR-p-DHT→AR-p-DHT-2	314.2469136	24.0220259	3.92E-07
mRNA-CycD-eIF4E-40S+60S→mRNA-CycD-eIF4E-40S-60S	315	33.07913118	6.12E-06
Her2-2-sPAcP→Her2-2-p+sPAcP	315.3580247	27.72708307	3.46E-08
Her2-2-p-Grb2+Sos→Her2-2-p-Grb2-Sos	317.6296296	59.31322182	0.000564659
Raf-p-Pase1→Raf+Pase1	320.1481481	22.06922878	4.43E-16
MEK-p+Raf-p→MEK-p-Raf-p	320.8518519	38.42622892	8.15E-06
ERK-pp-AP1→ERK-pp+AP1	324.1975309	20.51158446	9.84E-12
Act-Akt-Pase7→Akt+Pase7	324.9382716	60.78799269	0.000162736
Ras-GTP-GAP→Ras-GTP+GAP	325.4691358	51.16406287	2.46E-06
Ar-cPAcP→cPAcP+40S+60S+mRNA-cPAcP	326.0246914	23.44263701	1.16E-07
ERK-p+MEK-pp→ERK-p-MEK-pp	327.0493827	27.88791575	1.06E-17
Grb2+Sos→Grb2-Sos	327.1604938	39.71532216	3.42E-08
Her2-2+sPAcP→Her2-2-sPAcP	327.3580247	30.45193479	1.27E-09
MEK-pp-Pase2→MEK-pp+Pase2	328.1604938	37.76265872	3.48E-10
ERK-pp-ETS→ERK-pp+ETS	328.1728395	23.58267358	8.85E-16
AR-DHT→AR-p-DHT	328.5061728	16.30271123	3.19E-20
MEK-pp+Pase2→MEK-pp-Pase2	329.691358	37.64590232	6.26E-11
g-PSA+AR-p-DHT-AR-p→g-PSA-AR-p-DHT-AR-p	330.1975309	14.39799925	6.17E-34
ERK-pp+AP1→ERK-pp-AP1	330.8518519	16.21597311	2.17E-17
ERK-MEK-pp→ERK-p+MEK-pp	332.1975309	27.89075245	1.29E-22
ERK-pp-ETS→ERK-pp+ETS-p	332.7654321	19.2636008	5.16E-28

continued on next page

continued from previous page

Reaction	mean rank	st. dev rank	p-value
mRNA-cPAcP-eIF4E-40S+60S→mRNA-cPAcP-eIF4E-40S-60S	333.5802469	39.66926425	1.46E-11
g-cPAcP-RNAp→g-cPAcP+RNAp	334.0617284	23.57449206	2.04E-22
g-sPAcP+RNAp→g-sPAcP-RNAp	335.2345679	21.4478534	1.11E-25
ERK-pp-AP1→ERK-pp+AP1-p	336.5679012	12.72150447	1.21E-42
AR+DHT→AR-DHT	336.6419753	24.96844324	2.66E-19
Her2-2-p-Shc-p-Grb2-Sos+Ras-GDP→Her2-2-p-Shc-p-Grb2-Sos-Ras-GDP	338.382716	29.15859015	1.19E-20
ERK-pp+ETS→ERK-pp-ETS	339.3580247	12.66611069	3.29E-31
g-PSA+AR-p-DHT-2→g-PSA-AR-p-DHT-2	339.4567901	16.73725918	1.08E-33
PtdIns3+PTEN→PtdIns3-PTEN	339.5432099	29.20838114	4.00E-19
Shc-p→Shc	340.962963	15.7600779	2.34E-36
mRNA-cPAcP-eIF4E+40S→mRNA-cPAcP-eIF4E-40S	341.1234568	42.40786004	3.29E-14
Her2-2-p-Shc-p-Grb2-Sos+ERK-pp→Her2-2-p-Shc-p-Grb2-Sos-ERK-pp	341.345679	37.42933508	6.94E-17
Her2-2-p-cPAcP→Her2-2+cPAcP	343.345679	24.48144981	1.80E-16
sPAcP→sPAcP-e	343.4691358	28.577317	1.82E-18
Her2-2-p+Grb2-Sos→Her2-2-p-Grb2-Sos	344.4074074	37.79657755	5.91E-14
Her2-2-p-Grb2-Sos+Ras-GDP→Her2-2-p-Grb2-Sos-Ras-GDP	344.7654321	20.4743634	5.62E-29
mRNA-sPAcP+eIF4E→mRNA-sPAcP-eIF4E	345.8641975	35.32643812	2.14E-23
Her2-2-p-Shc-p-Grb2+Sos→Her2-2-p-Shc-p-Grb2-Sos	346.345679	34.82597178	2.39E-23
g-sPAcP-RNAp→g-sPAcP+RNAp+mRNA-sPAcP	346.7530864	40.97365413	1.22E-18
PtdIns3-PTEN→PtdIns2+PTEN	348.4814815	30.11608933	6.77E-23
AR-p-DHT+AR-p→AR-p-DHT-AR-p	348.7530864	16.13929669	2.31E-37
Ras-GTP-GAP→Ras-GDP+GAP	349.2222222	19.54340294	1.37E-41
Her2-2-p+cPAcP→Her2-2-p-cPAcP	351.1358025	29.00637813	1.58E-26
Her2-2-p-Grb2→Her2-2-p+Grb2	351.308642	21.45407774	8.46E-36
g-cPAcP+RNAp→g-cPAcP-RNAp	351.7037037	6.308996347	7.31E-52
mRNA-cPAcP+eIF4E→mRNA-cPAcP-eIF4E	353.0493827	36.12296635	8.29E-22
Her2-2-p-Shc-p-Grb2→Her2-2-p-Shc-p+Grb2	354.4567901	25.81287016	2.21E-40
Her2-2-p-Shc-p+Grb2→Her2-2-p-Shc-p-Grb2	355.7530864	23.27666671	3.34E-44
Raf-p+Pase1→Raf-p-Pase1	356.7037037	16.62646157	1.53E-46
Her2-2-p-Grb2-Sos+ERK-pp→Her2-2-p-Grb2-Sos-ERK-pp	357.3333333	19.06625276	1.92E-52
ERK+MEK-pp→ERK-MEK-pp	363.691358	5.008314417	2.09E-35
ERK-p-Pase3→ERK+Pase3	364	14.42986285	3.04E-33
Ras-GTP+Raf→Ras-GTP-Raf	364.6296296	9.591200954	1.32E-59
Her2-2-p-Shc-p→Her2-2-p+Shc-p	364.9382716	16.77372302	1.25E-61
PtdIns2-Act-PI3K→PtdIns3+Act-PI3K	365.8395062	20.72790082	1.52E-39
Ras-GTP+GAP→Ras-GTP-GAP	366.2592593	7.643337584	4.45E-60
Her2-2-p+Shc→Her2-2-p-Shc	366.5555556	19.83800321	6.79E-53
AR-HSP→AR+HSP	373.962963	5.201171826	5.92E-56
