# Supplementary material for: Analysis of the Molecular Networks in Androgen Dependent and Independent Prostate Cancer Revealed Fragile and Robust Subsystems
Source: PLoS One. 2010 Jan 28;5(1):e8864. doi: 10.1371/journal.pone.0008864 (PMC2812491; doi:10.1371/journal.pone.0008864)
Supplement: Table S5 — Statistically significant sensitivity differences between AI and AD LNCaP clones. Negative changes in the mean rank denote interactions that were more sensitive in AI versus AD cells. (0.02 MB PDF) [file pone.0008864.s005.pdf]

Table S5: Statistically significant sensitivity differences between AI and AD LNCaP clones. Negative changes in the mean rank denote interactions that were more sensitive in AI versus AD cells.

Reaction	p-value	△ mean rank
**rank(C-33) - rank(C-81)**		
Her2-2→Her2-2-p	2.41E-14	-87.2565701
cPAcP→[]	2.97E-11	-60.05830583
Her2-2-p-cPAcP→Her2-2+cPAcP	1.11E-16	-46.32587703
Ar-PSA→PSA+40S+60S+mRNA-PSA	9.08E-14	-45.08495294
mRNA-cPAcP-eIF4E-40S-60S→mRNA-cPAcP-eIF4E-40S+60S	2.10E-05	-43.08984232
Ar-cPAcP→cPAcP+40S+60S+mRNA-cPAcP	8.00E-12	-40.0840973
MEK-pp-Pase2→MEK-p+Pase2	0.005304844	29.80992544
2*Her2-2-p+cPAcP-2→2Her2-2-p-cPAcP-2	2.34E-11	34.46632441
ERK-p+Pase3→ERK-p-Pase3	2.30E-14	35.77753331
cPAcP-4→2*cPAcP-2	1.74E-08	44.76995477
AR-p-DHT-2+g-sPAcP→AR-p-DHT-2-g-sPAcP	1.14E-14	44.86273072
2*cPAcP-2→cPAcP-4	9.22E-09	45.43002078
AR-p-DHT-AR-p+g-sPAcP→AR-p-DHT-AR-p-g-sPAcP	8.30E-10	46.80723628
g-PSA-AR-p-DHT-2-RNAp→g-PSA-AR-p-DHT-2+RNAp+mRNA-PSA	0	66.02713605
**rank(C-33) - rank(C-51)**		
Her2-2-p-cPAcP→Her2-2+cPAcP	0.000173573	-11.53809276
2Her2-2-p-cPAcP-2→2*Her2-2+cPAcP-2	0.005962433	-10.58394997
Her2-2-p+cPAcP→Her2-2-p-cPAcP	0.039330397	-8.560083377
4Her2-2-p-cPAcP-4→4*Her2-2+cPAcP-4	0.030899498	-7.999478895
Ar-PSA→PSA+40S+60S+mRNA-PSA	0.0163948	-7.574361647
g-CycD-RNAp→g-CycD+RNAp+mRNA-CycD	0.008798024	6.428035435
Her2-2-p-Grb2-Sos→Her2-2-p-Grb2+Sos	0.03404035	10.82219906
4*Her2-2-p+cPAcP-4→4Her2-2-p-cPAcP-4	7.57E-05	14.0875456
g-PSA-AR-p-DHT-2-RNAp→g-PSA-AR-p-DHT-2+RNAp+mRNA-PSA	0.006362828	14.14799375
**rank(C-51) - rank(C-81)**		
Her2-2→Her2-2-p	2.28E-10	-73.21936322
cPAcP→[]	7.80E-07	-45.71812865
mRNA-cPAcP-eIF4E-40S-60S→mRNA-cPAcP-eIF4E-40S+60S	1.06E-05	-44.09317739
Ar-cPAcP→cPAcP+40S+60S+mRNA-cPAcP	1.22E-10	-37.77205978
Ar-PSA→PSA+40S+60S+mRNA-PSA	3.96E-10	-37.51059129
Her2-2-p-cPAcP→Her2-2+cPAcP	1.73E-10	-34.78778428

continued on next page

continued from previous page

Reaction	p-value	△ mean rank
Her2-2-p-Shc-p-Grb2-Sos-ERK-pp→Her2-2-p-Shc-p-Grb2+Sos+ERK-pp	2.51E-09	-24.51604938
AR-p-DHT-AR-p-g-sPAcP→AR-p-DHT-AR-p+g-sPAcP	1.72E-05	25.18258609
4Her2-2-p-cPAcP-4→4*Her2-2+cPAcP-4	4.10E-05	29.57764782
ERK-p+Pase3→ERK-p-Pase3	1.68E-10	30.16075374
2*Her2-2-p+cPAcP-2→2Her2-2-p-cPAcP-2	3.71E-12	36.44704353
AR-p-DHT-2+g-sPAcP→AR-p-DHT-2-g-sPAcP	1.81E-13	42.27784276
cPAcP-4→2*cPAcP-2	4.06E-08	44.55932424
2*cPAcP-2→cPAcP-4	3.21E-08	44.70318389
AR-p-DHT-AR-p+g-sPAcP→AR-p-DHT-AR-p-g-sPAcP	2.05E-09	46.47654321
g-PSA-AR-p-DHT-2-RNAp→g-PSA-AR-p-DHT-2+RNAp+mRNA-PSA	5.55E-16	51.8791423
